# Appraisal of Machine Learning Approach to Drifts and Stopes Damage Monitoring in Underground Mines

**DOI:** 10.3390/s26216860

**Published:** 2026-10-29

**Authors:** Kayode Komolafe, Kun Zhang, Peter Kolapo, Conteh Alpha

**Affiliations:** 1 Mining Engineering Department, Colorado School of Mines, Golden, CO 80401, USA; 2 Mining Engineering Department, University of Jos, Plateau 930105, Nigeria; 3 Earth Excavation Mechanics Institute, Colorado School of Mines, Golden, CO 80401, USA; 4 Kilduff Underground Engineering, Engineering Department, Denver, CO 80202, USA

**Keywords:** deep mining, safety, deformation, structural integrity, predictive models, algorithms

## Abstract

The quest to address the escalating demand for minerals, driven by technological advancements across various sectors, has necessitated the expansion of deep underground mining operations. As mines extend deeper, the safety risks associated with such environments have become increasingly critical. To mitigate these risks, a comprehensive assessment of underground mine workings is essential to evaluate potential damage and prevent failures in access drifts and stopes. However, the use of conventional ground monitoring instrumentation for deformation data acquisition and subsequent analysis has often proved insufficient for anticipating hazards, especially in complex geological formations within deep underground mines. Recent advancements in big data structuring and the development of intelligent algorithms have significantly improved the ability to address complex challenges, particularly in managing underground space infrastructure. Machine learning (ML) methods such as supervised learning, reinforcement learning, and unsupervised techniques have proven to be powerful tools for monitoring and damage detection in underground environments. This paper provides a focused review of ML applications for the evaluation and monitoring of damage in underground drifts and stopes, highlighting the impact of these technologies on enhancing safety and operational efficiency in deep mining operations. These techniques enable real-time data processing, pattern recognition, and predictive analytics, offering new insights into structural integrity and early warning systems for potential failures. The outcomes derived from the application of various ML techniques indicate that, in some cases, these methods may yield results that are comparable to, or even surpass, those obtained from well-established empirical approaches.

## 1. Introduction

The potential of unexploited deep mineral reserves remains a driving force for the global mining industry. This drive is fueled by industrial demand, the need for sustainable resource development, and the growing importance of critical minerals to the global economy. As a result, prospecting, exploration, and extraction activities are increasingly being extended into these resource-rich depths [1]. Increasing mine depth may significantly affect underground infrastructure, with the most challenging issue being the safety of workers, mine equipment, and underground facilities. One of the major practices to ensure the safety of both life and equipment in underground excavations is to maintain the stability of the excavated surfaces. On some occasions, the unexpected transition from a controlled ground failure process to a brittle failure process that may impact underground drift and working stopes is the hallmark of many fatal accidents and disasters in the underground mines. A drift is a crucial infrastructure in underground mining, characterized as a horizontal or slightly inclined underpass. It is strategically created to provide safe and efficient access to ore bodies. It enables the transportation of excavated materials and equipment, and ensures proper ventilation throughout the mine. Stoping is a process of extracting valuable ore from an ore body in underground mines from the designated areas, leaving behind open spaces known as stopes. These openings are carefully designed to balance the efficient recovery of ore, workings and operations of the mines with the stability of the surrounding rock structure. Depending on the mining method and safety considerations, stopes may be left open, filled with waste material, or reinforced to prevent collapse and ensure the safety of ongoing mining operations. The stability and orientation of drifts and stopes are vital, as they not only support the overall operational flow but also play a key role in maintaining the safety and structural integrity of the excavated openings underground.

The focus on drifts and stopes deserves justification. Drifts serve as the principal access and haulage routes, so any loss of serviceability interrupts production and compromises egress. Stopes are the largest and most transient openings in a mine, excavated in ground of variable quality and frequently carrying only light support. Between them, the two excavation classes appear to account for most ground-related incidents and personnel exposure hours in deep metal mines, and their damage mechanisms, driven by mining-induced stress change, blast damage accumulation and time-dependent rock mass behavior, differ from those of civil tunnels in ways that fixed-interval inspection is poorly placed to capture.

The aim of this appraisal is to examine critically how ML techniques have been applied to the evaluation and monitoring of damage in underground drifts and stopes, and to establish where these methods stand relative to conventional practice.

Drifts and stopes also differ in how they fail and, consequently, in how they are monitored. Drift damage typically appears as roof sag, rib spalling and floor heave, with squeezing or strain bursting under high stress; the parameters of interest are convergence, roof-to-floor closure [2] and support load, captured by extensometers, convergence stations and load cells, and the published ML applications lean toward time-series learners such as LSTM variants. Stope damage instead develops as hangingwall slough, overbreak and dilution, driven by blast damage and stress redistribution around large spans; cavity monitoring surveys, microseismic arrays and stability graph parameters supply the data, and classification learners such as random forest, ANN and SVM dominate. The review is organized along this division, which sets its boundary and limits repetition between the two contexts.

Deep underground mines are safely practicable only through understanding and management of rock mass failure mechanisms. Yielding and caving are common failure mechanisms around the underground openings. Yielded rock is a condition where the rock mass has undergone some level of deformation, such as bending or buckling, without experiencing complete failure or caving. However, an increase in the excavation span or radius can lead to the immediate collapse of the rock around the excavation. Yielded rock may have become permanently deformed, weakened and unable to return to its original state of stress. The underground design engineers take into consideration parameters such as the physical and mechanical characteristics of the rock mass when designing the drifts, stopes and other infrastructure. It is crucial to consider the behavior of rock masses when designing underground infrastructure that involves excavation. The stability of these excavations, according to [3], depends on the physico-mechanical properties of the rock mass, the discontinuity parameters [4], the groundwater condition, and the induced stresses. Accurately estimating or predicting the stability of underground excavations is challenging due to the complexity of the physical system and the extensive effort required to determine the input data related to geotechnical parameters [5,6,7,8,9,10]. These parameters are generally determined using experimental and in situ measurement techniques. Many reviewed publications on ML for ground movement monitoring focused on multiple applications without a specific focus on drifts and stopes damage monitoring. Therefore, this paper reviews the use of ML methods for the evaluation and monitoring of damage around underground drifts and stopes. The remainder of the paper is organized as follows: Section 2 sets out the literature search and scope; Section 3 examines the maintenance of stope and drift integrity in underground mines; Section 4 reviews conventional monitoring techniques; Section 5 outlines the ML techniques they feed; Section 6 covers damage mechanisms and ML applications to prediction; Section 7 draws together current challenges and future directions, and Section 8 concludes.

### Background of the Study

The quest to address the growing demand for critical minerals used in electric vehicles, electronics and semiconductors, aerospace, and renewable energy technologies has increased the need for deep underground mining. This shift is driven by declining ore reserves and the depletion of high-grade deposits in many conventional open-pit mines. Besides, the environmental issues associated with open-pit mines such as land degradation, water pollution, and habitat destruction [11,12] have underscored the need for more sustainable mining practices. This has led to a shift towards deep underground mining to address geological uncertainties, socio-environmental factors [13], geotechnical constraints, and economic viability. Deep mining operations face the significant influence of in-situ stresses, which emerge as the primary factor driving underground deformation and failure [14,15]. Geotechnical engineers cannot underestimate the influence of stress redistributions around excavations as drifts are being driven through a disturbed rock mass, leading to the development of working stopes. Deep mining can result in additional geomechanical challenges [16] because rock mass behavior changes and becomes more difficult to anticipate as in-situ stress increases relative to depth increase, which upsurges operational risks. A good geotechnical knowledge of stope and drift damage mechanisms, together with the ability to capture concealed deformation patterns, can help geotechnical engineers identify anomalies and damage. This supports safety assessment and condition evaluation of underground structures, particularly in complex geological environments. Variable rock quality due to irregular formations like faults, dykes, and folds can complicate drift driving and stoping operations, disrupt drifts’ excavation paths and stability. These discontinuities may result in tragic accidents, equipment damage, loss of production, and high cost of stope and drift rehabilitation whenever their equilibrium status is altered. These conditions require adaptive support strategies to maintain safety and operational efficiency. Mining activities can induce new fractures or activate existing ones, further disrupting the in-situ stress state of the rock mass. As mining operations progress, assessing the risk of roof falls becomes increasingly important. However, traditional ground monitoring instruments used for deformation data acquisition and analysis have proven inefficient in predicting roof falls, particularly in complex geotechnical environments found in deep underground mines. These limitations introduce uncertainty into the design and implementation of effective support systems.

## 2. Review Search and Scope

This appraisal is guided by four research questions. RQ1: Which ML techniques have been applied to damage evaluation and monitoring in underground drifts and stopes? RQ2: Which sensing systems and data streams feed these techniques? RQ3: How does the reported performance compare with conventional empirical and instrumentation-based approaches? RQ4: What gaps remain between demonstrated capability and routine mine-site deployment?

Peer-reviewed literature was identified through Scopus, Web of Science and Google Scholar using search strings that combined machine learning terms (machine learning, deep learning, neural network, random forest, support vector machine) with excavation and damage terms (drift, stope, underground mine, ground control, damage, deformation, monitoring). The search covered January 2000 to June 2026 and was supplemented by manual screening of reference lists. Studies were included if they applied an ML technique to damage evaluation or monitoring of underground mine openings, or if their methods were directly transferable from comparable underground works. Studies on surface mining, rock slopes, or purely civil infrastructure without transferable methods were excluded, as were non-peer-reviewed sources.

Where a retained study does not deal natively with drifts and stopes, it is flagged as transferable at the point of citation. RQ1 is addressed in Section 5 and Section 6, RQ2 in Section 4 (Table 1) and the remote sensing discussion in Section 5, RQ3 in Section 6.2 (Table 2), and RQ4 in Section 7 and Section 8.

## 3. Maintaining Stope and Drifts Integrity in Underground Mines

Over the years, researchers have focused on studying and analyzing the potential hazards of rock falls, particularly in underground mining methods such as longhole open stoping and longwall mining [17]. A stope is defined as an artificial space created when a desired quantity of ore is removed from the surrounding rock in an underground mine through mechanical excavation. The method of stoping varies depending on factors like the rock’s geomechanical properties, deposit size, and ore grade. Drifts, often compared to the arteries of a mine [18], are typically driven parallel to the orebody. They provide access to stopes, facilitate ore transportation, and serve as passages for workers and machinery. Effective design and proactive monitoring of these critical infrastructures are essential for maximizing ore recovery while maintaining the structural integrity of the surrounding rock. Rock falls in hard rock underground mines are a leading cause of both fatal and nonfatal injuries. As mines extend deeper, the risks associated with underground safety increase, along with production costs. Over time, conventional ground monitoring instrumentation for deformation has not effectively minimized ground failure risks nor supported efficient resource extraction in challenging underground environments [19].

**Figure 1:**
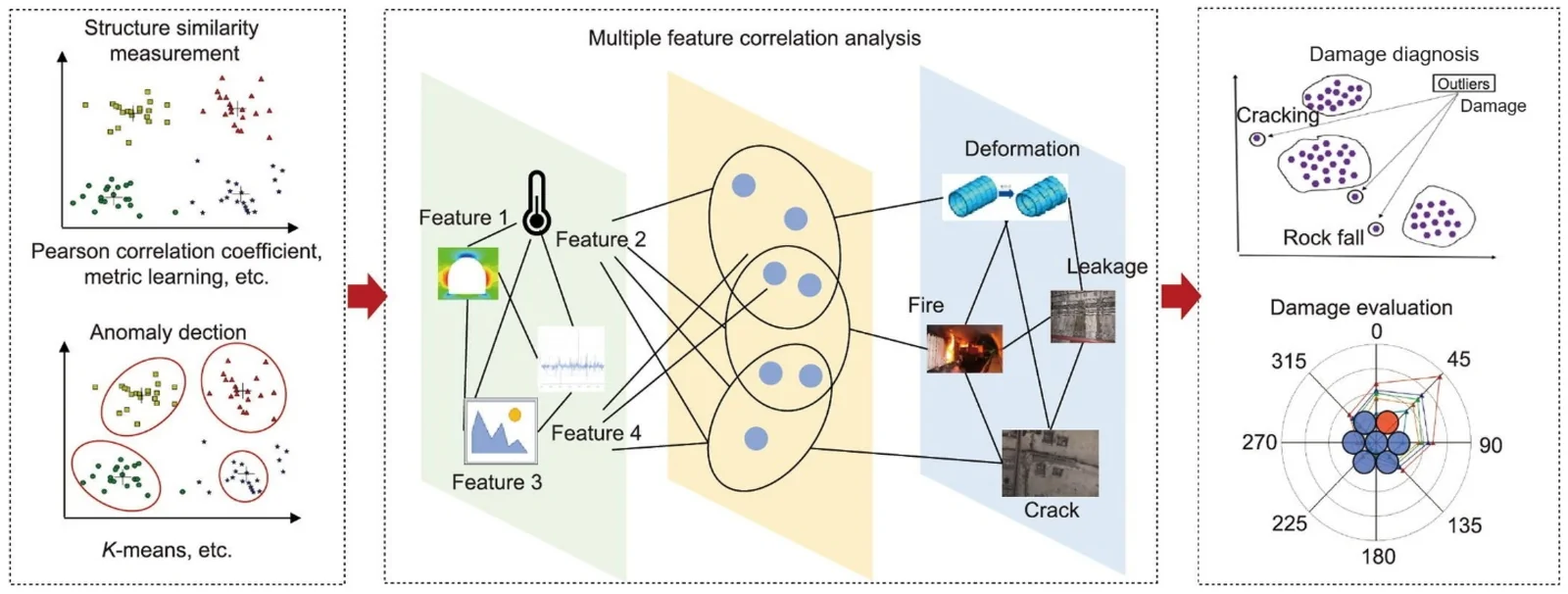
Discerning Hidden Pattern for Dynamic Anomaly Detection [21].

Recent research areas in the maintenance of underground infrastructure integrity are focused on damage localization and assessment [20,21]. Accurate damage assessment of the underground infrastructure plays a key role in ground support optimization efforts for maintenance, ensuring safety, and sustainable production. Also, locating the source and the mode of damage with precision would assist the geomechanics engineers to generate predictive models capable of forecasting further damage by simulating the form and stages of damage. However, the continual growth in data volume constitutes a huge challenge in consistent use of physical-based principles. Furthermore, ascertaining the physical model’s accuracy may prove challenging, given the potential variability and significant impact of data quality [21]. Recent advances using data-driven methods [22] in damage detection [23,24] and assessment of underground infrastructures have provided a series of solutions to these problems. ML methods could be integrated with analytical models and numerical simulations to estimate the physical properties and predict current structural conditions. Analysis of large volumes of data was made possible using ML to locate the source and the mode of structural damage with precision, as well as the discovery of hidden correlations and sensitive features to assess the underground mine’s infrastructural integrity. Identifying hidden deformation patterns supports real-time detection of structural anomalies in underground mines. This is achieved by examining similarities in observed behavior and tracking deviations from expected movement trends. As illustrated in Figure 1, through thorough analysis of multiple features and correlation analysis, the model captures complex interdependencies among variables to detect damage such as rockfalls, cracking, and other structural outliers. This integrated approach supports accurate damage diagnosis and evaluation. Improved reliability of monitoring systems for drifts and stopes can be achieved through this method. The autoregressive model [25], state-space model [26,27,28], and the ANN [29,30], are among the few data-driven approaches used by researchers in recent times for damage assessment [31,32].

## 4. Conventional Techniques for Drift and Stope Monitoring in Underground Mining

Ground monitoring in underground mines draws on a family of complementary instruments as summarized in Table 1. Displacement-based devices such as extensometers and convergence stations, stress and load measurement systems, and geophysical methods such as microseismic arrays each capture a different symptom of rock mass response, and each produces data of different frequency, dimensionality and noise character. These characteristics determine which ML techniques suit which data stream, which is why the instruments are introduced before their pairing with learning methods. Displacement-based devices are considered first.

### 4.1. Contact-Based Sensing

Extensometers have been used for decades in underground mining to monitor and measure rock displacement. This technology represents a conventional method for capturing displacement data, which is crucial for understanding the extent of rock mass deformation in underground environments. When materials are excavated, the equilibrium stress state within the rock is disturbed. This may lead to deformation as the rock adjusts to new stress conditions. This deformation can manifest as rock movement, convergence, or even fracturing.

The effectiveness of extensometers in measuring rock displacement depends on several factors, including the geometry of mine openings, the initial in-situ stress state, and the mechanical properties of the rock. By accurately measuring these parameters, extensometers provide important information on rock displacement during and after excavation. However, their performance can be affected by poor grouting at the anchor points and sensitivity to surrounding environmental conditions. In addition, their measurements are limited to specific locations, leading to the development of alternative methods such as fiber optic gauges.

### 4.2. Optical Sensing

Fiber optic strain gauges (FBG), though not a new technology, have seen significant advancements in recent years, positioning them as a superior option for deformation measurements. These gauges utilize unique optical properties of fibers to deliver reliable strain data, making them indispensable in geotechnical and structural monitoring. Among various types of fiber optic strain gauges, fiber Bragg grating is the most commonly used in geomechanics. Originally developed for telecommunications, FBG technology was adapted for various fields through the introduction of periodic variations in the refractive index of the fiber core. FBG has extensive applications across industries, including structural, aerospace, and geotechnical monitoring, where accurate and real-time [33] deformation measurements are vital for ensuring structural integrity and safety. These sensors offer advantages such as immunity to electromagnetic interference, high sensitivity, reliability, and the ability to transmit signals over long distances without degradation. Additionally, up to 100 FBG sensors can be integrated into a single optical fiber through multiplexing, enabling efficient monitoring of large-scale systems.

However, FBG sensors also exhibit certain disadvantages. The technology behind FBG sensors remains relatively complex, requiring specialized knowledge and expertise for installation, calibration, and interpretation of data. They are inherently fragile, making them susceptible to damage if mishandled or subjected to excessive strain. Another common limitation is the lack of an effective data acquisition system for accurately recording important magnetic field characteristics linked to anomalies or departures from normal patterns [34]. These characteristics include amplitude, intensity, and phase. Inadequate estimation of these magnetic field characteristics could lead to incomplete or distorted signal interpretation. This may reduce the sensitivity and reliability of FBG sensors in identifying micro-damages or early warning signs of structural failure. This limitation underscores the need for improved data-driven methods to enhance the resolution and accuracy of FBG-based rock damage detection systems.

A further complication is cross-sensitivity: the Bragg wavelength shifts with strain and temperature at once, at roughly 1.2 pm per microstrain against about 10 pm per degree Celsius, so an uncompensated reading conflates thermal and mechanical effects. Underground, where ventilation air and blasting generate sharp thermal transients, this error can swamp the strain signal of interest. Machine learning enters the FBG chain at three points. Learning-based peak detection resolves overlapping spectra in multiplexed arrays, trained networks suppress noise without requiring a clean reference signal, and classification models separate strain from temperature in single-grating or multi-array configurations [35,36] What remains after demodulation, denoising and discrimination is a residual signal in which anomalies may indicate micro-damage, which is where the damage identification applications reviewed below take over.

Reference [37] recommended that exploring machine learning and artificial intelligence algorithms can provide promising solutions for predicting the intensity and positioning of magnetic fields using arrays of FBGs while reducing signal processing complexity. Reference [38] further emphasized that applying conventional neural networks for signal processing assessment can enhance measurement accuracy while simplifying signal processing complexity associated with arrays of FBGs. Accurate interpretations of extensive historical deformation data have necessitated improved methods for monitoring and measuring deformation in underground spaces. This need has driven the applications of ML in stope and drift monitoring. This advanced technique can process large volumes of data more efficiently and identify subtle patterns that may be difficult to detect using conventional methods. As a result, it can improve the accuracy of deformation prediction and support safer underground operations.

### 4.3. Geophysical Sensing

Geophysical methods are close to the instrumentation family. Microseismic arrays locate and characterize fracturing events remote from any gauge, and acoustic emission systems capture the higher-frequency end of the same process at laboratory and near-field scale. Their data streams, event catalogues and waveforms rather than point displacements, suit sequence and classification learners, and recent work on rockburst location prediction from microseismic monitoring [39] indicates the direction this pairing is taking. Distributed fiber optic sensing, which shares hardware with the optical family but delivers spatially continuous strain profiles along boreholes and drives, sits between the two groups and has so far seen little mining application [40,41].

**Table 1:** Sensing families for drift and stope monitoring: strengths, limitations and typical ML pairings.

Sensing Family	Representative Instruments	Strengths	Limitations	Typical ML Pairing
Contact-based	Extensometers, convergence stations, load cells	Direct measurement; point precision; mine-hardened hardware	Point coverage only; anchor and grouting sensitivity; manual reading in many mines	Regression and time-series models on displacement and load records
Optical	FBG arrays, distributed fiber optic sensing	Multiplexed, immune to electromagnetic interference; high strain resolution	Fragile; temperature-strain cross-sensitivity; specialist interrogation equipment	Learning-based demodulation, denoising, strain-temperature discrimination
Remote	LiDAR, photogrammetry, InSAR (surface expression)	Full-field geometry without installed sensors; repeatable scans	Line-of-sight, dust and lighting constraints; no positioning underground without SLAM	CNN and point cloud deep learning for crack, overbreak and convergence detection [42].
Geophysical	Microseismic arrays, acoustic emission	Volumetric coverage; senses fracturing remote from any gauge	Location uncertainty; noisy catalogues; array design constraints	Event classification, location prediction and early warning models

## 5. Overview of ML Techniques for Monitoring Underground Structure Integrity

At the architectural level, the techniques reviewed here differ more than the shared ML label suggests. A multilayer perceptron passes a fixed-length input vector through fully connected layers of weighted sums and nonlinear activations, trained by backpropagation. Random forest instead aggregates the votes of decorrelated decision trees grown on bootstrap samples, so its output is a committee decision rather than a fitted surface. A support vector machine separates classes with a maximum-margin hyperplane and handles nonlinearity through kernel functions. LSTM networks add gated memory cells to the recurrent architecture, which lets the model retain or discard information across long monitoring sequences. Convolutional networks exploit spatial locality through shared filters, which is a property suited to imagery and rasterized point clouds.

**Figure 2:**
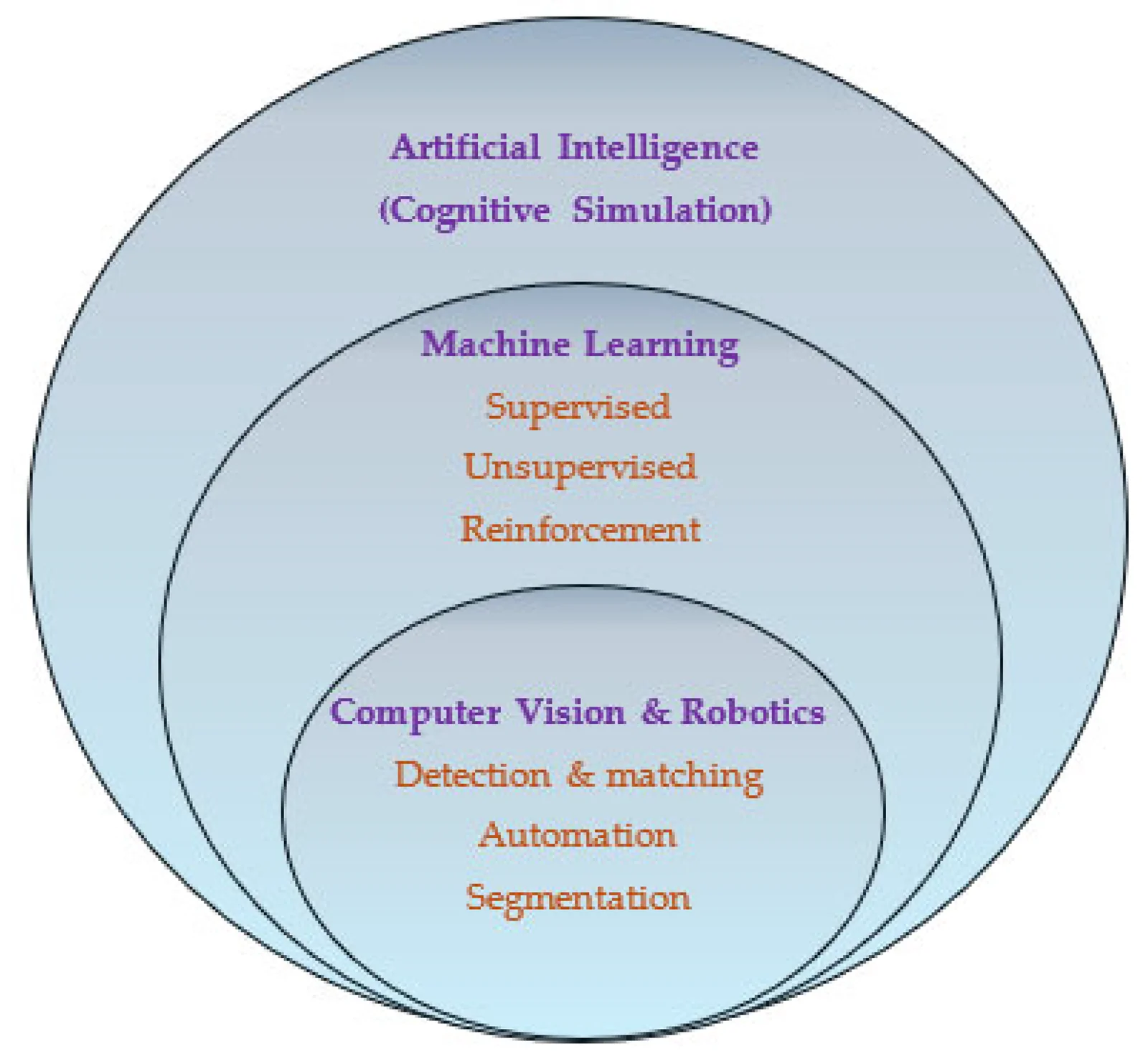
Synergy of Computational Intelligence.

**Figure 3:**
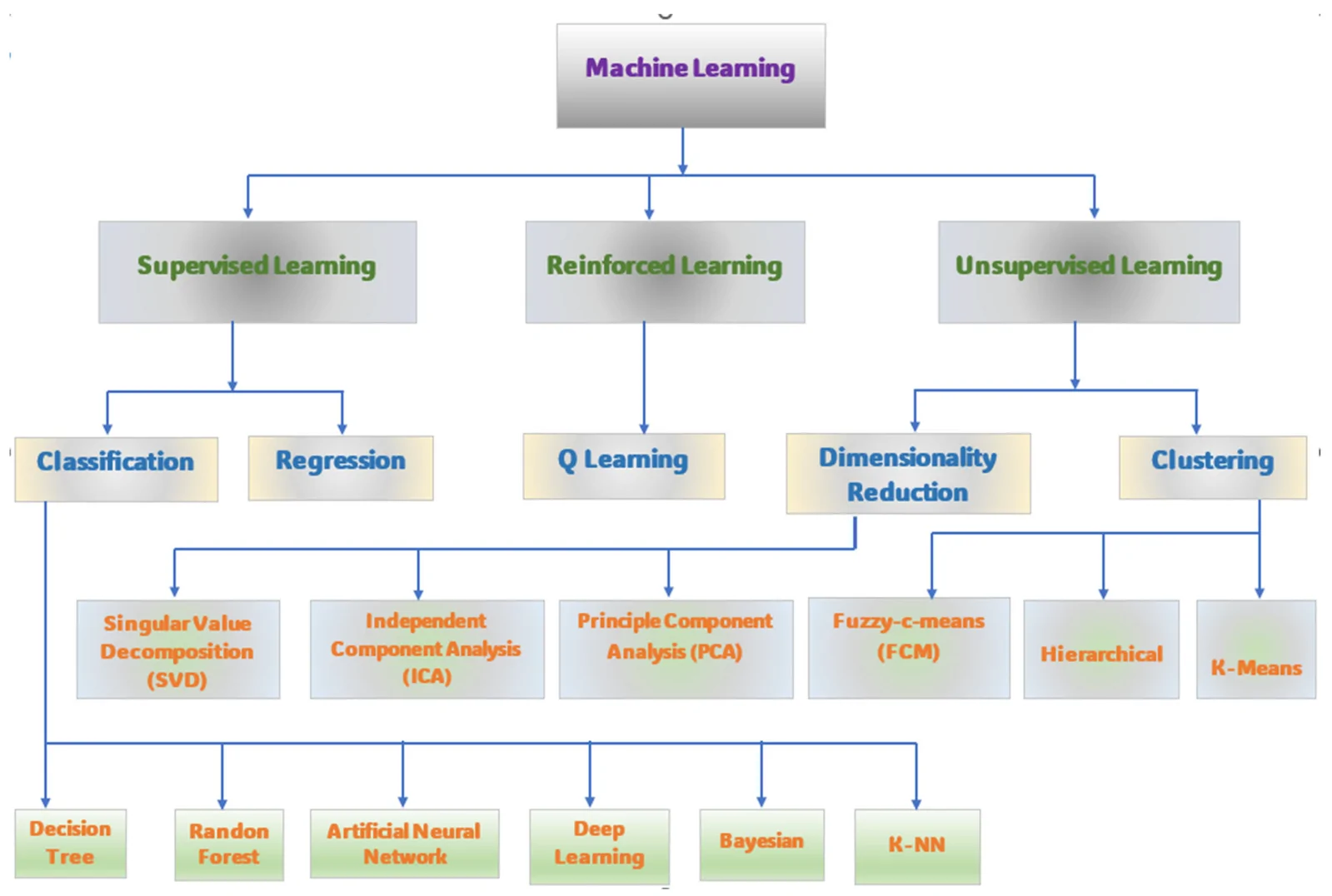
Classes of ML.

The synergy of computational intelligence techniques provides a strong basis for monitoring the structural condition of underground excavations. It incorporates artificial intelligence methods that simulate expert reasoning for decision-making in complex geotechnical environments. As illustrated in Figure 2, this synergy has catalyzed advances in big data analytics, robotics, and automation, which are now widely adopted in rock mechanics for the management of underground infrastructure. ML is a field that enables computers to learn and improve from experience without explicit programming. It leverages high computational power to process vast amounts of data using supervised, unsupervised, and reinforcement learning algorithms. These models enable adaptive pattern recognition, damage classification, and predictive diagnostics. Further enhanced by developments in computer vision [43] and robotics, such systems support detection, segmentation, and automation tasks essential for anomaly localization and inspection in challenging or inaccessible underground environments. Reference [44] classified ML mainly into supervised learning, unsupervised learning, and reinforcement learning. Many subsets of this classification, as shown in Figure 3, have been applied and proven to be a veritable tool in geotechnical and rock mechanics engineering.

**Figure 4:**
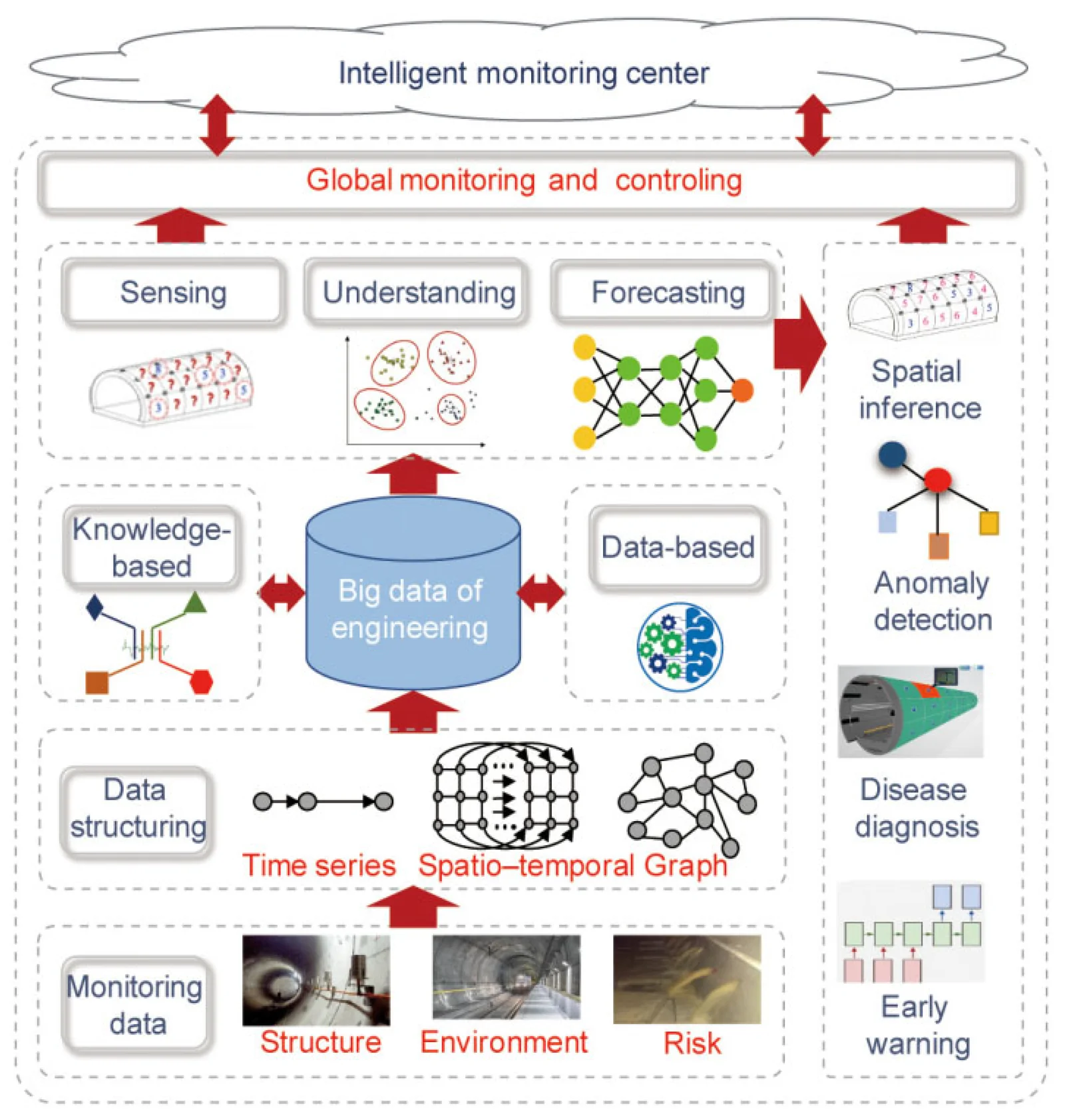
Harnessing monitoring data for underground Structure integrity [21].

The ML model is developed using data collected from monitoring instrumentation sensors and external factors such as geological structures, environmental conditions, and risk categories. Raw data are organized into structured formats according to their characteristics, including time series, spatio-temporal data, and graph data. By applying both domain knowledge-based methods and data-driven approaches, the geotechnical analysis of these extensive datasets uncovers the mechanisms behind rock mass damage. As illustrated in Figure 4, Reference [21] assert that by integrating information from diverse sources, the ML data structure achieves a holistic understanding of the strength and stability of underground infrastructure. This comprehensive approach not only improves anomaly detection but also provides valuable information about potential risks. This aids informed decision-making for managing underground structures. ML allows machines to acquire knowledge from historical data or experiences without explicit programming. It relies on extensive sets of structured and semi-structured data, empowering machine learning models to produce precise results or predictions based on the provided information.

### Remote Sensing

Equipment such as built-in sensors, interferometric synthetic aperture radar (InSAR), ground-penetrating radar (GPR), imaging, and laser scanning are used to acquire a large volume of data. These data are comprehensible and decodable by ML and computer vision-based techniques [43,45]. The technology involves the deployment of various sensors and built-in ground instrumentation equipment. Hence, the visualization and manipulation of the analysis, facilitating the assessment and decision-making processes (e.g., seismic-event prediction and classification of rock mass damage), can be achieved by establishing an application layer in the form of a graphical user interface [46]. For instance, ground instrumentation techniques such as InSAR can accurately measure tunnelling-induced changes [47] and support 4D mapping and environmental monitoring. The technology provides near-real-time measurements with millimeter-level accuracy [48]. This has been a veritable assessment tool for landslide deformation [48] and building damage [49] induced by tunneling [50,51] or drift driving. Reference [52] demonstrated using a CNN-based framework to detect the ground deformation induced by coal mining and tunnelling by employing high-resolution InSAR images (5 m/pixel) and UK velocity maps of 2015–2019.

Recent applications have moved from imagery to point clouds [53]. Graph-based deep learning frameworks now segment lining cracks directly from LiDAR point clouds [53,54,55,56,57], and SLAM-supported scanning permits crack assessment from a moving platform. Three-dimensional crack detection without color information has also been demonstrated [57,58,59], which matters underground where lighting cannot be relied on.

It revealed areas where information precision is dependent on manual inspection and objective expert interpretation [46]. A good geotechnical knowledge of damage mechanisms and capturing concealed patterns can help the system to identify anomalies and damages, providing safety analysis and state evaluation for the structure.

## 6. Mechanism of Stope Damage in Underground Mines

The inherent properties of the rock mass and boundary conditions in deep mines are the main contributors to the increased risks leading to disasters [15,60] like yielding and caving during deep mining operations. Acquiring comprehensive baseline data and considering the current state of the rock mass structure helps in predicting future tendencies. Also, continuous monitoring of the system with early warning capabilities ensures safety, thereby building a unified intelligent monitoring system [21]. The concentration of stress within the surrounding rock mass has the potential to cause both damage and failure, leading to collapse [61,62].

Reference [63] established that excavation unloading can trigger progressive slabbing failure and could activate internal structural planes. When excavating underground, it is anticipated that the rock mass surrounding the opening will experience radial displacement, and the ground surface will undergo subsidence, depending on the properties of the rock mass and its proximity to the surface. These effects are a natural consequence of the removal of material and the redistribution of stresses in the surrounding rock mass. Proper monitoring and management of these effects are crucial to ensure the stability and safety of the excavation and its surroundings. The failure process starts with a failure initiation phase, progresses through a propagation phase and ends in rock mass collapse [64]. Warning signs, which may be intensified by various triggering factors [65,66], can escalate from localized damage to regional-scale failure if not promptly addressed. Reference [67] quantified the hazards of rock fall and rock spalling based on the ML method of artificial neural network (ANN). In the study, sixteen geological, mining, technical and monitoring-related parameters were used to generate roof fall predisposition index and excavation maintainability index. Brittleness is one such parameter that is essential for understanding rock behavior under varying loading conditions in high-stress deep underground mines. Brittleness reflects a rock’s tendency to fracture or break suddenly with little plastic deformation. This means that brittle rocks are more likely to fail suddenly and sometimes catastrophically when stressed beyond their strength limit. This property is particularly critical in deep mining environments where high stress levels can lead to sudden fracturing or spalling, causing immediate rock deterioration. Rock masses such as sandstone, kimberlite, kaolinite, shale, and other weak rock types often exhibit poor geomechanical behavior similar to that of over-consolidated clay [68]. In brittle rock conditions, failure can result in extensive fracturing around mining excavations. Key concerns in such cases include the support and control of fractured rock, mine infrastructure design, and the selection of suitable stoping methods [69]. By considering rock brittleness, engineers can take early steps to minimize the risk of uncontrolled rock deterioration. This approach also helps to limit rock displacement and deformation around underground openings. As a result, safer excavated spaces can be maintained within the underground environment. Reference [70] asserted that the strength and deformation parameters of the rock masses are the basis for analyzing their behavior and judging the stability of the engineering project. As ML continues to advance, its application in predicting rock properties, particularly in complex rock masses, is becoming increasingly important. These predictive models support more reliable assessment of the integrity of underground structures.

### 6.1. ML Approach to Prediction of Mechanical Properties of Rock

The development of infrastructure in deep hard-rock underground mines often spans various rock types or geotechnical domains, each characterized by distinct geomechanical properties. Accurate determination of these properties is important for the design of underground infrastructure. It helps to maintain worker safety and support sustainable production throughout the life of the mine. Conducting these assessments in the field or laboratory can be tedious and costly, particularly when time is a critical factor. As a result, researchers have developed empirical and mathematical models to indirectly estimate certain mechanical properties of rock, using simple parameters that can be measured either in situ or in the laboratory [71].

Rock brittleness is one of the critical parameters influencing rockmass stability and failure, especially during or after excavation due to stress redistribution. Understanding rock brittleness helps in designing effective support systems and preventing catastrophic failures in mining and civil engineering projects. Rock strength parameters that influence brittleness properties such as tensile strength, unit weight of the rock and UCS were used as input by [72] to predict rock brittleness. A fuzzy inference system (FIS) was used to obtain fuzzy predictions, which were compared to the nonlinear regression analysis of the measured values [73].

ANN, which is one of the classifications of supervised learning, was used by [74] to predict the brittleness of sedimentary, metamorphic and igneous rocks. The input parameters used are dynamic Poisson’s ratio, Lame’s constant, dynamic Young’s modulus, unit weight, and P- and S- wave velocities. The ANN-generated model does not take other parameters influencing the strength properties of the rock into consideration, yet the model demonstrated a better fit compared to the existing study and the MLR. The limitations imposed by input parameters are one of the reasons why the model may not be fully accepted for practical applications in the geotechnical fields.

Reference [75]’s prediction model using particle swarm optimization (PSO) and imperialist competitive algorithm (ICA) for rock brittleness showed a better performance compared to [74] with respect to the coefficient of correlation and predicted rock brittleness. Reference [75] chose wave velocity, density, Schmidt hammer rebound number and point load index as the input variables, while the brittleness index was treated as the output value. The inadequacies with regard to the precision of these models have hindered their widespread application. Therefore, it is essential to engage advanced ML techniques to develop more accurate and robust models for predicting the rock brittleness index.

### 6.2. Specific Application of ML for Enhanced Design and Predicting Stope Stability

Persistent assessment of stope stability is a continual challenge due to the inherent complexities of rock masses, such as anisotropy, heterogeneity, and the presence of discontinuities [76]. These factors significantly complicate the prediction of stability, making it difficult to account for variations in rock behavior. The rockmass properties and the stope geometry play critical roles in the design of open stopes in underground mines. The presence of fissures and large discontinuities in the rock mass surrounding the stope dictates how stable the stope would be. It is well noted that mining activities induce both new fractures and activate existing ones within the rock mass, disrupting the in-situ stress state. Therefore, assessing the risk associated with stope instability becomes increasingly crucial as mining operations advance in underground mines.

The Mathews stability graph design method, first developed in 1981, is a key empirical approach used by mining and geotechnical engineers for designing stopes. This method utilizes the relationship between the stability number (N) and the hydraulic radius (HR) to determine appropriate span dimensions and support, based on the rock mass conditions associated with the stope. Reference [77] defined N using the following equations:

N = Q′ · *A* · *B* · *C* (1)

where [78]

(2) $$Q^{\prime }\;=\frac{RQD}{J_{n}}\cdot \frac{J_{r}}{J_{a}}\;\mathrm{(modified Q value)}$$

*RQD* is the rock quality designation, and Jn is the rock mass Joint sets. Jr and Ja represent the joint roughness and the joint alteration. The values of A, which is the rock stress factor, B, which represents the joint orientation adjustment factor, and C, which is the surface orientation factor, are determined by the graphs developed by [76]. The Matthew’s stability graph [77] may not be suitable for stope design in deep underground mines, where high-stress conditions, seismic activity, and ground deformation are frequent, as these factors are not fully integrated into the method. Additionally, it lacks consideration for the dynamic nature of mining environments, as it does not incorporate real-time monitoring data, which is crucial for adapting to evolving ground conditions during excavation. These and many other limitations paved the way for the use of ML to enhance the design and predict the stability of the stope.

#### 6.2.1. Random Forest Algorithm for Stope Stability Prediction

A supervised learning method such as the random forest (RF) algorithm has been proven to be a versatile tool that can model the nonlinear relationship between inputs and output without statistical assumptions. The RF algorithm is an ensemble learning technique that builds multiple decision trees or groups of decision trees and combines their predictions to improve accuracy and reduce overfitting. The RF algorithm consists of a group of decision trees operating as a committee. Each decision tree is trained on a random subset of the training data and makes predictions independently. Each tree in the forest makes its own prediction for a given input. The classification predicted by the majority of the trees is chosen as the output; thus, the output is the value predicted by a larger number of decision trees. For regression tasks, the final output is the average of all the trees’ predictions. The RF algorithm has the ability to regulate key parameters that directly influence model performance, including adjusting specific parameters to control the performance of several decision trees. This flexibility enables effective control over overfitting and underfitting, ensuring more accurate and reliable predictions by balancing model complexity. Having more decision trees can enhance the model’s learning process and improve performance. However, increasing the number of estimators excessively may lead to issues such as slower processing times and a higher risk of overfitting the data [79]. Balancing the number of trees is crucial for optimal efficiency and accuracy.

**Figure 5:**
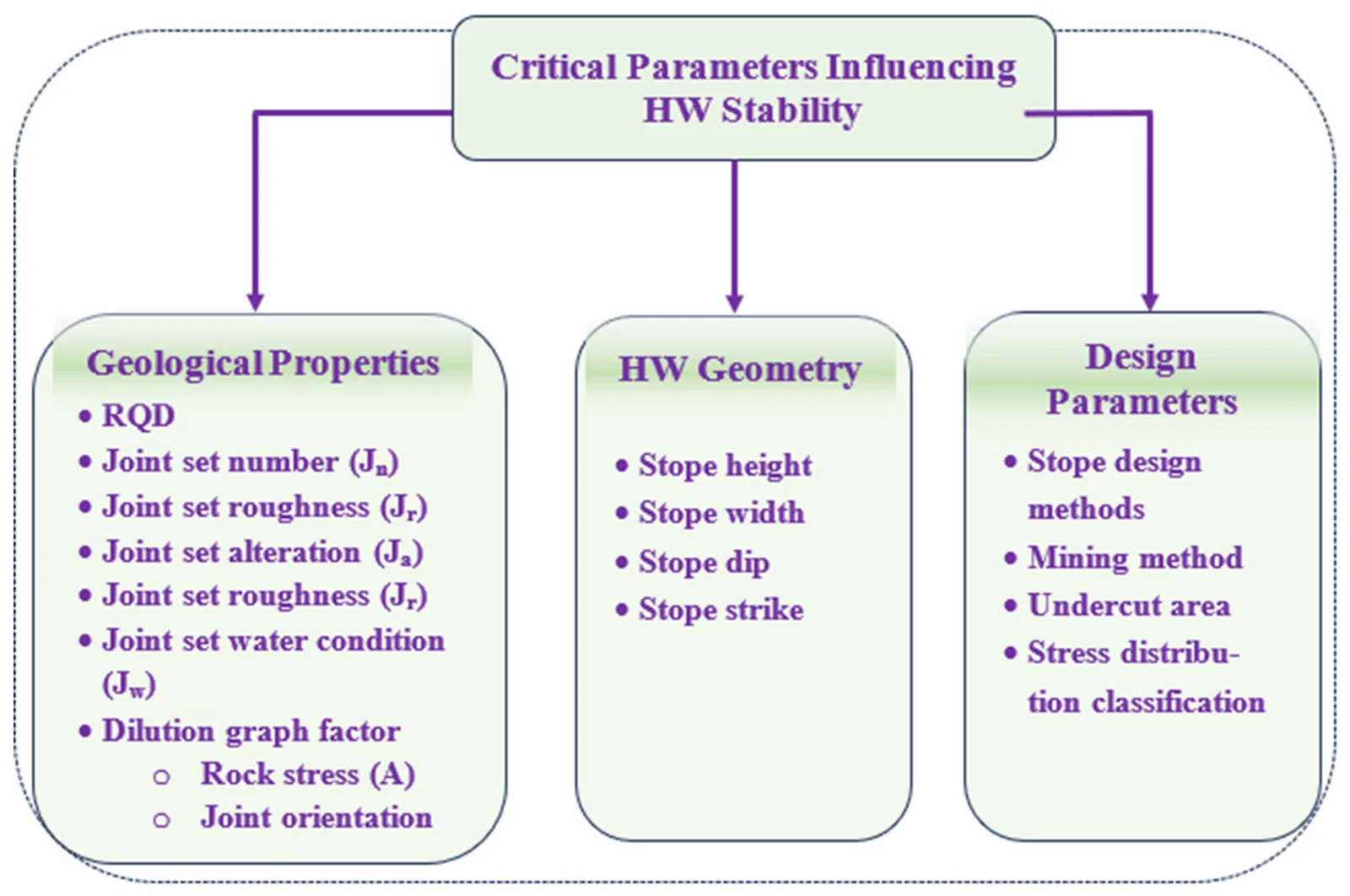
Parameters influencing the stability of an open stope (modified after [80].

Reference [80] analyzed 115 hanging wall (HW) cases to predict HW stability using the Random Forest (RF) algorithm. This algorithm offers an improved approach for predicting HW stability, as it overcomes the “generalization limitations” of the Mathews Stability Graph. Additionally, the RF algorithm is highly resilient to outliers and can effectively assess the relative importance of variables affecting hanging wall stability. As shown in Figure 5, major parameters influencing the stability of an open stope include the Rock Quality Designation (RQD), stope height, and stope design methods, among others. Reference [80] demonstrated that the RF model provides a quick and cost-effective method for estimating stope hanging wall stability, significantly reducing the time and resources required for stability assessments. Furthermore, its ability to identify the relative importance of influencing variables offers valuable insights for designing open stopes in underground mines.

Reference [79] input Potvin’s database into the RF algorithm using the sklearn library in Python, with the stability number (N) and hydraulic radius (HR) as inputs, and the output categories being stable, unstable, or caved. They used 20% of Potvin’s database to test the algorithm’s performance, while the remainder served as the training set. The study showed that RF performed better than logistic regression, with accuracies of 0.84 for the training dataset and 0.75 for the test dataset. The model achieved its best results in the “Stable” category by correctly predicting 17 out of 19 cases, while the 5-fold cross-validation produced an average accuracy of 0.71 with a standard deviation of ±0.06. The Potvin stability graph method simplifies stope stability classifications into two categories, which are stable and cave, separated by a transition zone. The limitations of the [79] model in predicting the transition between stable and unstable classes arise from similarities in N and HR values. This highlights an area for further investigation, which could be addressed by utilizing larger datasets and leveraging RF algorithms.

Stability classes in the stability graph space are gradational, and random forest models appear most error-prone precisely in the transition zone, where ensemble votes split nearly evenly and carry little confidence information unless calibrated. Reported accuracies also rest on random splits of modest databases, so some optimism in the headline figures is likely.

#### 6.2.2. Long-Short Term Memory for Drift Stress Prediction

Long short-term memory (LSTM) is a type of recurrent neural network (RNN) designed to capture both short-term and long-term dependencies in sequential data. This capability makes it suitable for geomechanical applications where temporal patterns and data sequences are important. Transfer convolutional neural networks (TCNNs) and LSTM networks are ML techniques that can effectively model and predict complex patterns associated with stress variations in underground space. In geomechanics, LSTMs can be used to model time-series data such as stress and strain variations, or deformation trends in drifts and stopes, enabling predictive insights into ground behavior under stress over time.

Reference [81] employed an LSTM to train microseismic data and the geomechanical parameters from a previously manually calibrated FLAC3D model to update a stress model at Garson Mine in Ontario, Canada. Two LSTM networks are trained for comparison, one to predict the principal stresses and the other to predict the six-component stress tensor. The predicted values demonstrated a high level of accuracy when compared with the manually computed values by the FLAC3D model [82]. LSTM offers a novel enhancement for mine-scale finite difference element models. This addresses the typically time-consuming and labor-intensive process that demands substantial engineering hours [83] and computational resources while updating a stress model [84]. TCNNs are better at learning spatial patterns in stress distribution, such as identifying high-stress zones or spatial relationships in rock formations, that can be applied to similar mining environments, saving significant time and computational resources in new projects.

Reference [85] demonstrated that spatial stress distribution across an underground roof can be effectively mapped using a TCNN model. Simulated data generated by the finite difference method was pretrained and calibrated to develop a predictive model. This model showed high accuracy when validated against structural health monitoring (SHM) data from a stress sensor installed in the roof of the Dongtan coal mine, extending up to 120 m ahead of the working face [84,85,86]. The study highlights that practical, site-specific predictions are achievable, even with limited field measurements, by integrating a physics-informed approach rather than relying solely on data-driven methods. This approach is also applicable to stope and drift stress prediction as mine workings and development progress.

TCNN models utilize past data to identify spatial-temporal patterns in stress distribution. It provides information about future stress behavior and aids in the optimization of support design and risk management in dynamic underground environments. LSTMs are effective at capturing temporal dependencies in sequential data, such as stress changes over time in an underground mine. This makes it usable for time-based predictions. Together, TCNNs can capture and transfer spatial stress distribution patterns within the rock mass. LSTMs can then model how these stresses change with time, providing a framework for predicting future stress behavior and improving support strategies as mining progresses. The use of advanced ML tools has improved the analysis of complex geotechnical problems. These methods are particularly useful in applications where both spatial rock structure and time-dependent stress changes must be considered. TCNNs can capture spatial stress distribution patterns, while LSTM networks can track how these stresses change over time. The combination of these approaches supports better predictive models for support design, safety improvement, and risk management in underground mining environments. This approach is invaluable for anticipating stress redistribution in mine workings to improve mine planning, and ensuring the stability of structures under changing geological conditions [87].

The recurrent models reviewed here share a structural dependence on record length and continuity. Underground monitoring streams are often short, gappy and irregularly sampled, conditions that appear to penalize LSTM architectures more than the civil tunnelling literature acknowledges. Where records span only a few loading cycles, simpler regressors may match recurrent networks at a far lower cost.

#### 6.2.3. ANN for Stope Stability Assessment

ANNs are computational systems designed to receive, interpret, and make decisions based on prior training. An ANN functions similarly to a biological neuron, “learning” by processing input samples paired with corresponding actions or outputs, thereby emulating the human brain’s approach to interpreting and responding to information. This technique is very useful in solving problems involving non-linear or highly dynamic relationships. ANNs have the ability to capture and model various complex interactions, which enable users to effectively represent phenomena that might otherwise be too difficult or impossible to explain through traditional methods [88]. This flexibility makes them a powerful tool for tackling intricate, data-driven challenges. Their range of adaptations includes back propagation neural network (BPNN), probabilistic NN (PNN), radial basis function NN (RBNN) and complementary NN (CMPNN), classified based on the different methods implemented to train the network.

These techniques have been extensively applied in rock mechanics, particularly for predicting rock properties. ANN establishes relationships between geotechnical and geometrical input parameters such as uniaxial compressive strength (UCS), density, Schmidt rebound hardness, and P- and S-wave velocities. These relationships are then used to predict the required output with improved accuracy. The relationship is built through learning from trained data, allowing the system to predict outcomes based on these input variables. This technique can process unlimited data, learning its characteristics to classify it with minimal error, making it effective for assessing open stope stability.

Reference [89] used an ANN to assess and predict the stability of stopes in vertical retreat mining (VRM), a sub-level stoping method. This approach was proposed as an alternative to the Mathews’ stability graph method [77] particularly for applications in deep mines that extend to depths of 1000 m or more. The use of the ANN provides a flexible approach for developing predictive models from historical data and observed patterns. This approach improves the understanding of stope stability under varying conditions encountered at such mining depths. Historical data were collected from 35 different open stopes in a Zinc mine, and artificial neural networks were used to process the data and classify the open stopes according to the stability regions of [77]. The researchers established that the networks were able to identify patterns and satisfactorily classify the studied open stopes. This machine learning technique showed strong performance in classifying open stopes as either stable or unstable. The model achieved an overall success rate of 82%, with an apparent error rate of 18%. There were three categories for classification: stable, transition, and unstable stopes. Internal validation produced a global success rate of 91% and an apparent error rate of 9%, which made the results more consistent. In contrast, the external validation showed a global success rate of 42% and an apparent error rate of 58%.

The external validation result of [89] deserves emphasis rather than a passing mention. A drop from 91% internal to 42% external success suggests a model fitted closely to the 35 stopes of one zinc mine rather than to transferable behavior, and it illustrates the small-sample overfitting risk that runs through much of the ANN literature reviewed here.

#### 6.2.4. Optimized ML Models for Ground Movement Prediction

**Figure 6:**
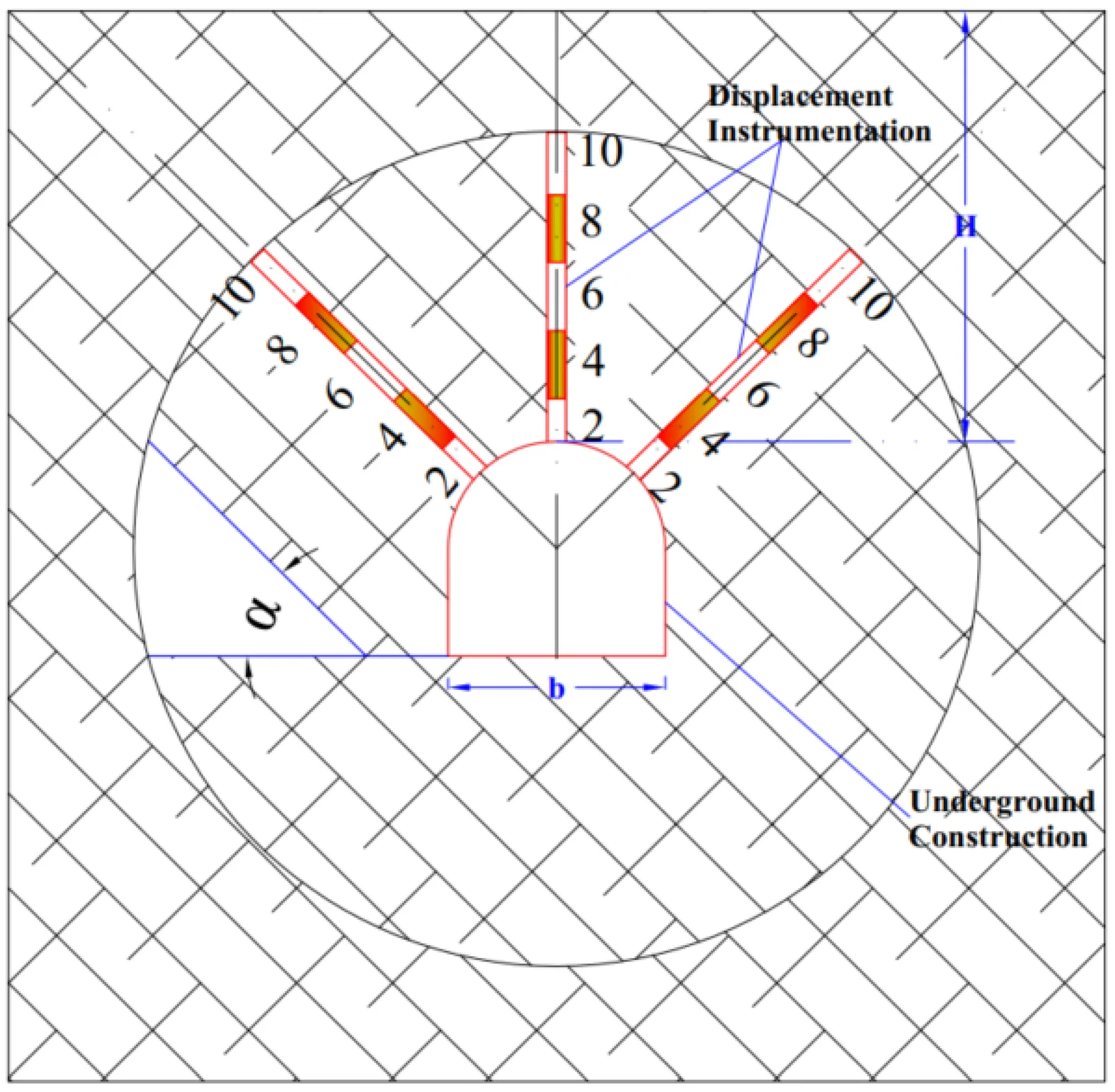
Instrumentation scheme for rock displacement in an underground mine.

A number of scholars have implemented optimized models as a solution to geomechanics problems in areas of ground movement prediction in underground mines. For instance, Reference [90] predicted rock displacement in underground mines using a Harris Hawks optimization algorithm (HHOA) based support vector machine (SVM) model (HHOA-SVM) and a Grasshopper optimization algorithm (GOA)-based SVM model (GOA-SVM). These improved machine learning algorithms were developed using datasets obtained from field measurement stations in underground mines. The datasets included observations of vertical rock mass displacement, rock properties, and monitoring time. Extensometers were used in the boreholes to monitor rock displacement, as shown in Figure 6, to acquire some necessary data. Reference [90] used rock properties, such as the depth of the tunnels (H), the angle of rock layers (α), anti-bending moment (Wc), the width of the tunnels (b), the tensile strength of rock layers (Rn), monitoring distance (*Lb*), and observation time (*t*), as the input data for the HHOA to predict rock displacement in the underground mines. Their findings revealed that *H*, *Lb*, *t,* and *α* are the most influential parameters for predicting rock displacement in underground mines and, by extension, in tunnels. This work confirmed the assertion of [15] that data-driven identification methods are less likely to be affected by changes in the external environment. The improved machine learning-based algorithms, which are developed with the datasets obtained from field measurements, produced more accurate results. As such, in the case of rock displacement, data obtained from the parameters that are directly related to the rockmass stability leads to more accurate identification of the micro-damages and hidden defects in underground space structures.

This is an improvement upon the traditional finite element-based evaluation methods. The data-driven identification methods allow for assessing structural behavior correlation from various angles and conceptualizing a feature set that captures early micro-damage. This approach is particularly useful in the exploration stage of HHO and non-linear feature extraction. The modified framework [21] shown in Figure 4 reveals that an adaptive anomaly detection approach can be developed. This can be achieved through extensive data mining, especially at the exploration stage of HHO for feature extraction. Its algorithm helps resolve the challenge of few-shot abnormal data samples by employing dimension reduction analysis on the source data [91]. It achieves this by deriving distinct damage-specific bases, uncovering concealed abnormal features. Moreover, HHO, as an improved ML-based model with adaptive anomaly detection, has become a veritable tool crucial for intelligently detecting concealed flaws and early-stage micro-damage in underground infrastructure [92].

Despite the effectiveness of HHO algorithms in solving various problems, especially when combined with other models to form hybrid systems, they have inherent limitations that require improvement. HHO is a random optimization algorithm and faces challenges such as maintaining population diversity and avoiding local optima, particularly with high-dimensional datasets. The initial population can significantly affect the algorithm’s performance; a poor initial population might result in longer convergence times or failure to find the optimal solution. Researchers are actively working to enhance the performance of HHOA in feature selection problems and to achieve better classification results.

Optimized hybrids invite a note of caution. Harris Hawks Optimization, like most metaheuristics, may converge prematurely to local optima in rugged hyperparameter landscapes, and the reported improvements over conventionally tuned models are seldom verified on held-out data. Without such benchmarks, the added complexity is difficult to justify. Table 2 draws the four algorithm families reviewed above into a single comparison. The figures are indicative ranges taken from the cited studies rather than universal values, and the ANN row may suggest how far small-sample results can travel outside their training mine.

**Table 2:** Side-by-side comparison of RF, LSTM, ANN and HHO-SVM for underground mining applications.

Algorithm	Typical Dataset Scale	Reported Performance (Examples)	Applicable Tasks	Core Limitations
Random forest	Tens to a few hundred tabular cases	80–95% classification accuracy in stope stability studies	Stope stability classification; feature importance ranking	No extrapolation beyond training range; transition-zone misclassification; importance biased to high-cardinality features
LSTM	Continuous monitoring streams; thousands of time steps	R2 above 0.9 for drift stress prediction where records are long	Time-series prediction of stress, convergence, seismic precursors	Degrades on short, gappy or irregular records; data-hungry; opaque without add-ons
ANN (MLP)	Small to medium tabular sets; often tens of cases	82% overall; 91% internal vs. 42% external validation [89]	Stope stability classes; rock property prediction.	Small-sample overfitting; ad hoc architecture selection; weak transfer to unseen mines
HHO-SVM	Small tabular sets	Gains of a few percent over untuned SVM reported	Displacement and roof pressure prediction with tuned hyperparameters [93]	Premature convergence to local optima; complexity rarely benchmarked against grid search

### 6.3. Physics-Informed Neural Networks and Physics-Embedded Feature Engineering

Purely data-driven models carry no obligation to respect mechanics, and the small databases common in mining make that freedom expensive. Physics-informed neural networks (PINNs) address the problem by embedding governing equations, yield criteria or boundary conditions in the loss function, so the network is penalized for physically implausible predictions [94]. A second, less formal route embeds physics in the features instead, deriving inputs from analytical solutions such as convergence confinement or Mindlin-type formulations before any learning takes place.

Demonstrations to date sit mostly in civil tunnelling. Elastic-plastic mesh-free PINN modelling of tunnelling-induced deformation [95]), PINN prediction of ground deformation from sparse field measurements [96] and settlement prediction beneath existing rail infrastructure all report usable accuracy from datasets that would starve a conventional network. Transfer to mine drifts and stopes appears promising but remains untested; blast loading, rapidly changing geometry and discontinuous rock masses violate assumptions the civil demonstrations lean on. That gap is arguably the clearest research opening this appraisal has identified, and it is taken up again in the conclusions.

Work published between 2024 and 2026 has begun to address several of the gaps identified above. Stability graph methods for stope design have been re-examined with ML on a database of roughly 980 unsupported stope cases [97], while intelligent models for locating rockburst from microseismic monitoring [98] and real-time warning schemes built on multi-feature microseismic parameters [99] have moved the field closer to operational early warning. Reviews of ML for overbreak and underbreak prediction [100] and for underground mine planning [101] point in the same direction. These studies appear to confirm the trend, noted throughout this appraisal, toward larger case databases and greater attention to transferability across sites.

## 7. Challenges and Future Directions

Several challenges recur across the studies reviewed. Databases are small and often imbalanced [102], with rockburst and stability compilations recycled between papers, and models are seldom tested outside the mine that trained them; the external validation collapse reported by [89] is likely to be representative rather than exceptional. Random data splits continue to inflate reported accuracy where monitoring records or multi-site databases are involved. On the sensing side, temperature-strain cross-sensitivity, data gaps from harsh operating conditions and the absence of underground positioning constrain the data streams that any learning method can work with. Interpretability tools remain under-used, which appears to slow acceptance among ground control engineers who must act on model output [103,104].

Future work may usefully move in five directions. Site-holdout validation deserves to become the reporting norm rather than the exception. Transfer learning across mines, so far barely explored in this literature, could ease the site-bound character of current models. Physics-informed approaches need extension from civil tunnels to blast-loaded, discontinuous rock masses. Distributed fiber optic sensing paired with learning-based interpretation offers spatially continuous strain coverage that point gauges cannot match. Finally, open benchmark databases, in the spirit of the large stope stability compilation of [97] would let methods be compared on common ground rather than on private datasets.

## 8. Conclusions

The integration of ML into underground drift and stope maintenance holds significant potential for enhancing safety in underground mining operations. Advances in data structuring, computer vision, and real-time monitoring are empowering ML applications to address complex geotechnical engineering challenges and improve the integrity of ground conditions. Problems previously deemed insurmountable with traditional instrumentation and monitoring methods are now being effectively addressed through ML innovations. Optimized machine learning models support the analysis of large datasets collected from different sensors in underground environments. By combining data from monitoring instruments, these models improve prediction accuracy and assist in identifying micro-damages and abnormalities in underground infrastructure. Despite challenges associated with isolated data and dimensionality reduction, these advanced ML techniques provide comprehensive insights into rock mass behavior, thereby enhancing safety and stability in underground mining environments. However, the scarcity of historical instrumentation data presents a significant challenge in developing ML methods that can accurately generate predictive models to enhance the safety and integrity of underground infrastructures. To counter this, employing advanced numerical modeling to simulate field conditions is recommended, especially in complex and hazardous drifts and stopes where access is limited. Future research should focus on advancing machine learning techniques to support intelligent sensing and early warning systems tailored for deep and complex underground mining environments, with the goal of ensuring operational safety. In addition, there is a need for robust models that can incorporate different historical datasets and improve through continuous validation. Such models can strengthen prediction reliability under varying geological conditions and support more dependable ground stability assessments in high-risk underground mines.

## Data Availability

The original contributions presented in this study are included in the article. Further inquiries can be directed to the corresponding author.
